# CRISPR/Cas9-mediated mutation on an insulin-like peptide encoding gene affects the growth of the ridgetail white prawn *Exopalaemon carinicauda*

**DOI:** 10.3389/fendo.2022.986491

**Published:** 2022-09-29

**Authors:** Yi Gao, Xiaojun Zhang, Jianbo Yuan, Chengsong Zhang, Shihao Li, Fuhua Li

**Affiliations:** ^1^ Chinese Academy of Sciences (CAS) and Shandong Province Key Laboratory of Experimental Marine Biology, Institute of Oceanology, Chinese Academy of Sciences, Qingdao, China; ^2^ School of Marine Science and Engineering, Qingdao Agricultural University, Qingdao, China; ^3^ Laboratory for Marine Biology and Biotechnology, Qingdao National Laboratory for Marine Science and Technology, Qingdao, China; ^4^ Center for Ocean Mega-Science, Chinese Academy of Sciences, Qingdao, China

**Keywords:** insulin-like peptide, *Exopalaemon carinicauda*, gene structure, expression profile, CRISPR/Cas9, RNA interference

## Abstract

Insulin-like peptides (ILPs) play key roles in animal growth, metabolism and reproduction in vertebrates. In crustaceans, one type of ILPs, insulin-like androgenic gland hormone (IAG) had been reported to be related to the sex differentiations. However, the function of other types of ILPs is rarely reported. Here, we identified another type of ILPs in the ridgetail white prawn *Exopalaemon carinicauda* (*EcILP*), which is an ortholog of *Drosophila melanogaster* ILP7. Sequence characterization and expression analyses showed that *EcILP* is similar to vertebrate insulin/IGFs and insect ILPs in its heterodimeric structure and expression profile. Using CRISPR/Cas9 genome editing technology, we generated *EcILP* knockout (KO) prawns. *EcILP*-KO individuals have a significant higher growth-inhibitory trait and mortality than those in the normal group. In addition, knockdown of *EcILP* by RNA interference (RNAi) resulted in slower growth rate and higher mortality. These results indicated that *EcILP* was an important growth regulator in *E. carinicauda*.

## Introduction

The insulin/insulin-like peptide superfamily is an ancient and evolutionarily conserved gene family that broadly regulates the metabolism across the Metazoa (1, 2). It is involved in controlling many aspects of life, such as growth, metabolism, reproduction, lifespan, resistance to stress conditions and immune activity (3–5). There are three major subgroups in this superfamily, including vertebrate insulin, insulin-like growth factors (IGFs) and protostomian insulin-like peptides (ILPs); there are also some other small subgroups, such as vertebrate relaxins and crustacean insulin-like androgenic gland (IAG) (6–8). Among protostomian, a large number of ILPs have been identified since the first ILP was discovered in *Bombyx mori* (silkworm) in 1984, known as bombyxin (9). Multiple ILPs have been reported in invertebrates and the numbers of ILPs are varied with species. For example, there are four ILPs in *Tribolium castaneum* (red flour beetle), seven in *Anopheles gambiae* (African malaria mosquito), eight in *Drosophila melanogaster* (fruit fly), ten in *Acyrthosiphon pisum* (pea aphid), and 38 in *B. mori* (1, 10, 11); in other species, such as the nematode, *Caenorhabditis elegans*, up to 40 distinct ILP genes have been identified (12).

In crustaceans, only one type of ILP has been discovered as the IAG, which is produced and secreted by the male-specific endocrine organ, androgenic gland (AG), was found to be a key regulator of male differentiation and maintenance of the male phenotype (13). IAG silencing resulted in complete functional sex reversal in giant river prawn *Macrobrachium rosenbergii* (14). Apart from IAG, no more ILPs have been reported in crustaceans until a second ILP was discovered in Eastern rock lobster *Sagmariasus verreauxi* (*Sv-ILP1*) in 2015 (15). *Sv-ILP1* is a *D. melanogaster* DILP7/relaxin ortholog, therefore it is known as DILP7/relaxin-like type of ILP. Broad expression of *Sv-ILP1* suggests that ILPs might have a role beyond masculinization in decapods (15). With the development of next-generation sequencing and bioinformatics analyses, four types of ILPs have been identified in crustaceans, including IAG, ILP7/relaxin, insulin-like peptide (similar to the *Drosophila* insulin-like peptides 1-6), and gonadulin (16, 17). Some crustaceans have been reported to have all four types of ILPs, while some species have only two to three types, possibly due to incomplete genomic and transcriptomic assembly and tissue-specific expression (17, 18). Although the identification of different ILPs confirms their existence in decapods, the functions of other types of ILPs apart from IAG, especially their effects on growth and metabolism, are still unclear.

The reported functions of the insulin superfamily are diverse in animals, including growth, metabolism, immunity and reproduction (10, 19, 20). In insects, the general consensus is that growth and development are regulated by the insulin signaling pathway through ILP receptor activation (21, 22). The function of ILPs has been extensively studied in insects, especially in *D. melanogaster* (10, 22). To date, eight ILPs (DILP 1-8) have been identified in *D. melanogaster* (23, 24), which can be divided into DILP1 produced by brain insulin-producing cells (IPCs) during pupal stages, DILP2-5 produced by IPCs in the embryo, DILP6 produced by the fat body, DILP7 produced by the neurosecretory cells different from IPCs, and DILP8 produced by the injured wing disk and epidermis (22, 24). These DILPs have showed obvious functional differentiation. Among them, seven ILPs (DILP1-7), especially DIL2, showed growth-promoting abilities (25, 26). DILP2 also can regulate lifespan (25), DILP5 and DILP6 are involved in body growth (27), DILP3 and DILP7 can affect appetite (28), and DILP8 mediates the signaling of the growth status of peripheral tissues to the brain complex (29, 30). The important roles of DILPs in controlling reproduction have also been demonstrated (31, 32).

Insulin/insulin-like peptides have a specific processing in their peptidyl maturation, they are synthesized as a signal peptide, B-chain, C-peptide and A-chain over an N-terminal to C-terminal (15). Post-translational modifications result in a removal of the signal peptide and the C-peptide, produce the mature hormone consisting of A and B chains. The conserved backbone is generated *via* six cysteine residues forming two interchains and one intrachain (within the A chain) disulphide bridges, which is a hallmark feature of insulin/insulin-like peptides (20, 33).

Considering the important role of ILPs in metazoans, it is of great interest to investigate the functions of ILPs in decapods. The ridgetail white prawn *Exopalaemon carinicauda* is an economically important crustacean and a decapod specie on which the CRISPR/Cas9 gene editing system has been achieved (34). In this study, we isolated and characterized a DILP7/relaxin-type ILP gene in *E. carinicauda*, and analyzed its function using the CRISPR/Cas9 gene editing and RNA interference (RNAi) technique.

## Materials and methods

### Screening of *EcILP* in *E. carinicauda*

Sequences annotated as ILP genes were collected from the *E. carinicauda* genome and transcriptome data in our laboratory (35). Using both the full-length and homogenous amino acid sequences from *D. melanogaster* and other arthropods as query sequences, BLASTP (https://blast.ncbi.nlm.nih.gov/Blast.cgi) search was performed to identify potential *EcILP* sequence with an E-value cutoff of 1e^-5^. The resulted hit sequence was then analyzed with both SMART (http://smart.embl.de/) and Pfam (https://pfam.xfam.org/) to ensure the presence of the insulin-like grown factor (ILGF) domain. The candidate *EcILP* sequence was submitted to ORF Finder (https://www.ncbi.nlm.nih.gov/orffinder/) to predict the region of open reading frame (ORF).

### Cloning of the full-length cDNA of *EcILP*

Total RNA was extracted from tissues using Trizol reagent (Invitrogen, USA) according to the manufacturer’s protocol. Then, the RNA was reverse transcribed to cDNA using a PrimeScript™ RT Reagent Kit (Takara Bio Inc., Japan). Gene-specific primers for *EcILP* were designed based on the ORF sequences by primer 5 software (**Table 1**). The full-length cDNA sequence of *EcILP* was amplified by polymerase chain reaction (PCR) using a high-fidelity DNA Polymerase (Vazyme, Nanjing, China). Each product was ligated into the pMD19-T cloning vector (TaKaRa, Japan) and transformed into DH5α competent cells (TransGen Biotec, Beijing, China). For each amplicon, approximately 30 independent colonies were sequenced at Sangon Biotech Co., Ltd. (Shanghai, China). The NCBI BLAST program was used to map sequenced ORF to the genomic data to identify exons and introns.

**Table 1:** The primers used in this paper.

Primers		Sequences
**Full-length primer**		
EcILP_FL_F		ACTGGAGGGCTGCTGATA
EcILP_FL_R		CTGCCGGAAACTGGATAA
**Quantitative primer**		
EcILP_Q_F		TTCCAGGACGGTTAGCGAGTG
EcILP_Q_R		CGAACCTTGTGCCCGATGG
18S_Q_F		TATACGCTAGTGGAGCTGGAA
18S_Q_R		GGGGAGGTAGTGACGAAAAAT
**Detection primer**		
EcILP_D_F		GCTAAACATTTTTCTGTGTAATCCAT
EcILP_D_R		ATCGCCAGATTGCTCACTTAC
**RNAi primer**		
ds-EcILP_F		TAATACGACTCACTATAGGGACTGGAGGGCTGCTGATA
ds-EcILP_R		TAATACGACTCACTATAGGGAGGGAGGGACGGTCTAAC

The forward and reverse primers are represented by F and R, respectively.

### Sequence characterization of *EcILP*

Amino acid sequences of *ILP* and *IAG* homologs from other arthropods were downloaded from NCBI database. Multiple sequences alignment and the neighbor-joining (NJ) phylogenetic tree with 1,000 bootstrap replicates were conducted by MEGA 6.0 (36). The sequence similarity scores for different ILP chains were obtained from NCBI BLASTP alignments. The deduced amino acid sequence of EcILP was analyzed with the ExPASy (Expert Protein Analysis System, https://www.expasy.org).

### *EcILP* gene expression in different larval stages and adult tissues

To analyze *EcILP* expression profiles, the early developmental samples of *E. carinicauda* were collected as the pool according to morphological classifications at six early developmental stages, including zygotes, blastocysts, nauplii, zoea, mysids, and postlarvae. Twelve healthy adult *E. carinicauda* with an average body length of 4.2 ± 0.4 cm were collected for tissue expression analysis. Seven different tissues including eyestalk, stomach, hepatopancreas, heart, epidermis, gill and muscle were dissected out and immediately frozen in liquid nitrogen for RNA extraction. Total RNA was extracted from the collected samples using Trizol reagent, and RNA was reverse transcribed to cDNA using the PrimeScript™ RT Reagent Kit according to the manufacturer’s protocols. Quantitative real-time PCR (qRT-PCR) was performed to analyze *EcILP* expression profile. 18S rRNA was used as the internal control. The primers of qRT-PCR are shown in **Table 1**. The experiment was repeated in triplicate for biological repeats, and each sample was run in triplicate on a qRT-PCR system for technical replicates. The relative expression levels were calculated using the comparative CT method formula (37) and shown as mean ± standard deviation (SD). One-way ANOVA was performed for significant differences (* *p* < 0.05) between values and tested using Duncan’s test with SPSS 16.0 (SPSS Inc., Chicago, IL, USA).

### sgRNA design and synthesis for *EcILP*

The single-stranded guide RNA (sgRNA) target sequence for *EcILP* was designed using the online tool CRISPRdirect (http://crispr.dbcls.jp/) (38). sgRNA was synthesized using the Thermo Scientific TranscriptAid T7 High Yield Transcription Kit (Thermo Fisher Scientific, USA). sgRNA concentration was assessed by Nanodrop 2000 (Thermo Fisher Scientific, USA) and quality was assessed by electrophoresis on 1% agarose gel.

### Microinjection of *E. carinicauda* fertilized eggs

Embryos microinjection protocol was performed as previously described (34, 39). Briefly, single-cell stage fertilized eggs were collected from the abdomen of gravid female prawns, which were cultivated with mature male prawns in aerated fresh seawater at 25°C. Purified sgRNA and Cas9 mRNA (Thermo Fisher Scientific, USA) were incubated and injected into *E. carinicauda* fertilized eggs at a concentration of 400 ng/ml for sgRNA and 450 ng/ml for Cas9 mRNA. The FemtoJet 4i-Injector microinjector (Eppendorf, Germany) and stereo microscope MMO4 micromanipulator (Narishige, Japan) were used for microinjection using standardized Femtotip II sterile microcapillaries (Eppendorf, Germany). All injected mixtures were prepared in RNase-free water containing 0.05% phenol red. The injection volume is approximately 0.5 nl. The control group was injected with an equal volume of RNase-free water containing 0.05% phenol red.

After microinjection, the embryos were transferred into a clean petri dishes with sterilized seawater and incubated at 100 rpm in a shaker at 27°C. Fresh clean seawater was changed twice a day. Mysis stage larvae were fed with artemia larvae and the juvenile stage prawns were fed commercial prawn pellet.

### Evaluation of CRISPR/Cas9 generated *EcILP*-injected embryos/individuals

The number of surviving embryos was counted at nauplius stage (72h), zoea stage (10 days) and mysis stage (15-16 days) after microinjection. A total of 240 nauplius stage and 150 zoea stage embryos were randomly selected from injected embryos to evaluate the efficiency of gene editing. After hatching, one leg of the juveniles was collected separately to detect indels and assess the efficiency of gene editing. Genomic DNA was extracted from each embryo and larval leg using a micro extraction method (39). The target fragment was amplified by PCR using a pair of detection primers that amplified a 410 bp fragment around the sgRNA-targeted sites (**Table 1**). Fragment amplified from wild type (WT) embryos was used as control. Sequence alignment was performed with MEGA 6.0 software, and sequencing chromatogram was viewed by Chromas software. Growth indicators of *EcILP*-KO individuals in adulthood, including body length and body weight, were detected over nine months. Growth indicators were measured individually for each mutant individual, and the results were shown as mean ± SD. Significance was analyzed between the two groups using Student’s t-test at the following significance levels: * *p* < 0.05. Statistical analyses were performed using SPSS 16.0.

### RNA interference (RNAi) of *EcILP* in adult *E. carinicauda*

Double-stranded RNA (dsRNA) was synthesized using a transcriptAid T7 High Yield Transcription Kit (Thermo Fisher Scientific, USA). Primers used for RNAi were designed based on the *EcILP* cDNA sequences (**Table 1**). Transcription templates were prepared by PCR using gene-specific primers with a T7 polymerase promoter sequence. Meanwhile, the dsRNA of the enhanced green fluorescent protein (EGFP) gene was synthesized as a negative control. The concentration and quality of synthesized dsRNA were detected using a NanodropTM 2000 Spectrophotometer (Thermo Fisher Scientific, USA) and 1.5% (w/v) agarose gel electrophoresis.

The optimum interference concentration of ds-EcILP was obtained by pre-experiment. After that, a 15 days RNAi experiment was performed to investigate whether *EcILP* was involved in the growth of *E. carinicauda*. A total of 225 healthy prawns (average weight 0.83g) were equally divided into three groups (PBS group, ds-EGFP group, and ds-EcILP group) and injected with 10 µL PBS, 10 µL ds-EGFP (1 μg/µL), and 10 µL ds-EcILP (1 μg/µL), respectively. Prawns were injected every four days. Body weight and mortality rate were recorded at the end of the experiment. The average weight gain and mortality rate were calculated according to a previously reported method (40). The results were shown as mean ± SD. One-way ANOVA for significant differences (* *p* < 0.05) was performed using Duncan’s test with SPSS 16.0.  

## Results

### Identification of *ILP* genes in *E. carinicauda*

**Figure 1:**
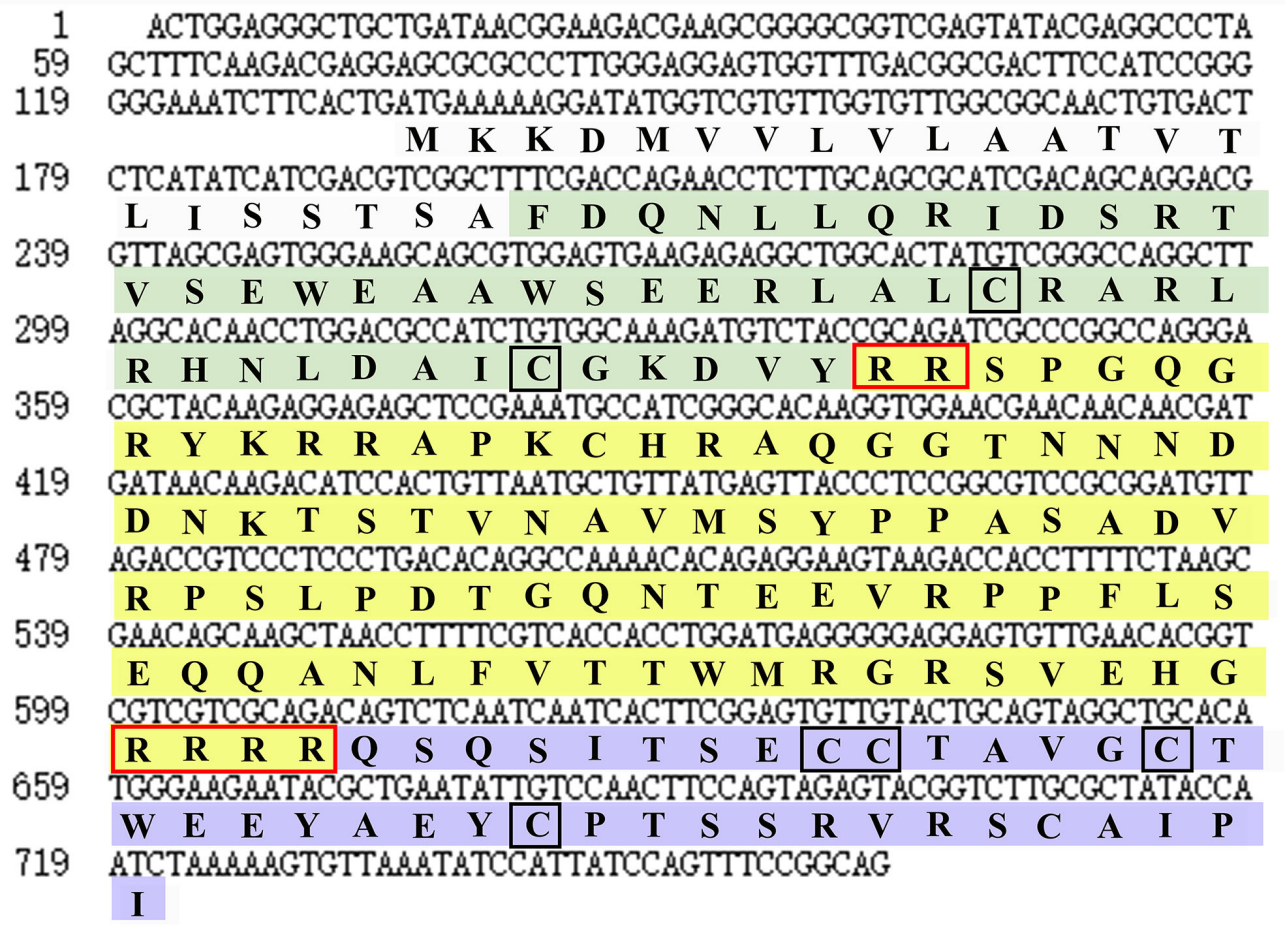
Nucleotide and deduced amino acid sequences of *EcILP*. Nucleotide and amino acid sequence of EcILP are shown in lowercase and uppercase, respectively. The sequence of B, C and A chains are stained in green, yellow and purple, respectively. Two cleavage sites and six cysteine residues are shown in red and black boxes, respectively.

**Figure 2:**
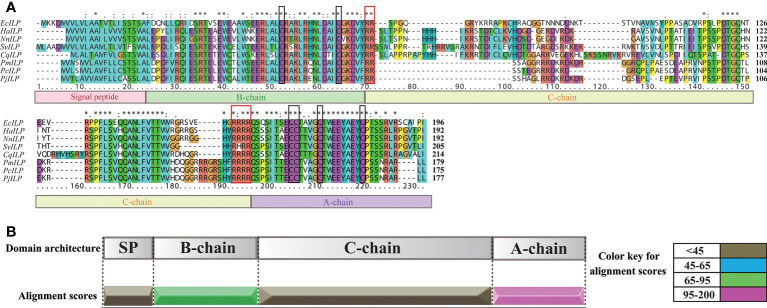
Multiple sequence alignment and sequence similarity scores of EcILP and other crustacean ILP proteins. **(A)** Multiple sequence alignment. Boxs under the sequences represent the signal peptide, B, C, A chains, respectively. Two cleavage sites and six conserved cysteine residues are shown in red and black boxes, respectively. The ILP sequence from *Exopalaemon carinicauda* (EcILP, in this paper), *Homarus americanus* (HaILP, XP_042233096.1), *Nephrops norvegicus* (NnILP, QBX89070.1), *Sagmariasus verreauxi* (SvILP, AIU40991.1), *Cherax quadricarinatus* (CvILP, AIU40992.1), *Penaeus monodon* (PmILP, XP_037788563.1), *Penaeus chinensis* (PcILP, XP_047482879.1) and *Penaeus japonicus* (PjILP, XP_042860749.1) were used for the alignment. Asterisks indicate fully conserved residues. **(B)** Sequence similarity score. Sequence similarity scores of different ILP chains are based on the multiple sequence alignment in **(A)**.

By screening the genomic and transcriptomic data, a gene that potentially encoding ILP (named *EcILP*) was identified in *E. carinicauda*. The full-length cDNA sequence (GenBank accession number: ON720329) of *EcILP* was amplified by reverse transcription-polymerase chain reaction (RT-PCR). Sequence analysis revealed that *EcILP* contains a 591 bp of open reading frame (ORF) encoding 196 amino acids (aa). The predicted molecular weight of EcILP proteins is 22.04 kDa, and the predicted pI value is 9.14. The key features of EcILP sequence structure are clearly apparent: an N-terminal signal peptide (22 aa); followed by a B-chain (46 aa) containing two cysteines; a C-chain (91 aa), flanked by RR and RRRR cleavage sites; and an A-chain (37 aa) containing one double and two single cysteines (**Figure 1**). The presence of two and four conserved cysteine residues in the putative B and A chains, respectively, were inferred to form disulphide bridges (C_51_–C_169_, C_63_–C_183_ and C_168_–C_174_), which are required for tertiary folding (**Figures 1**, **2A**). Sequence alignment revealed that the key sequence frameworks from different decapod species shared a high degree of sequence similarity, including B chain, A chain, six cysteine residues, and two cleavage sites (**Figures 2A, B**), but the similarity of cleavage sequences (signal peptide and C-chain) was low (**Figures 2A, B**).

**Figure 3:**
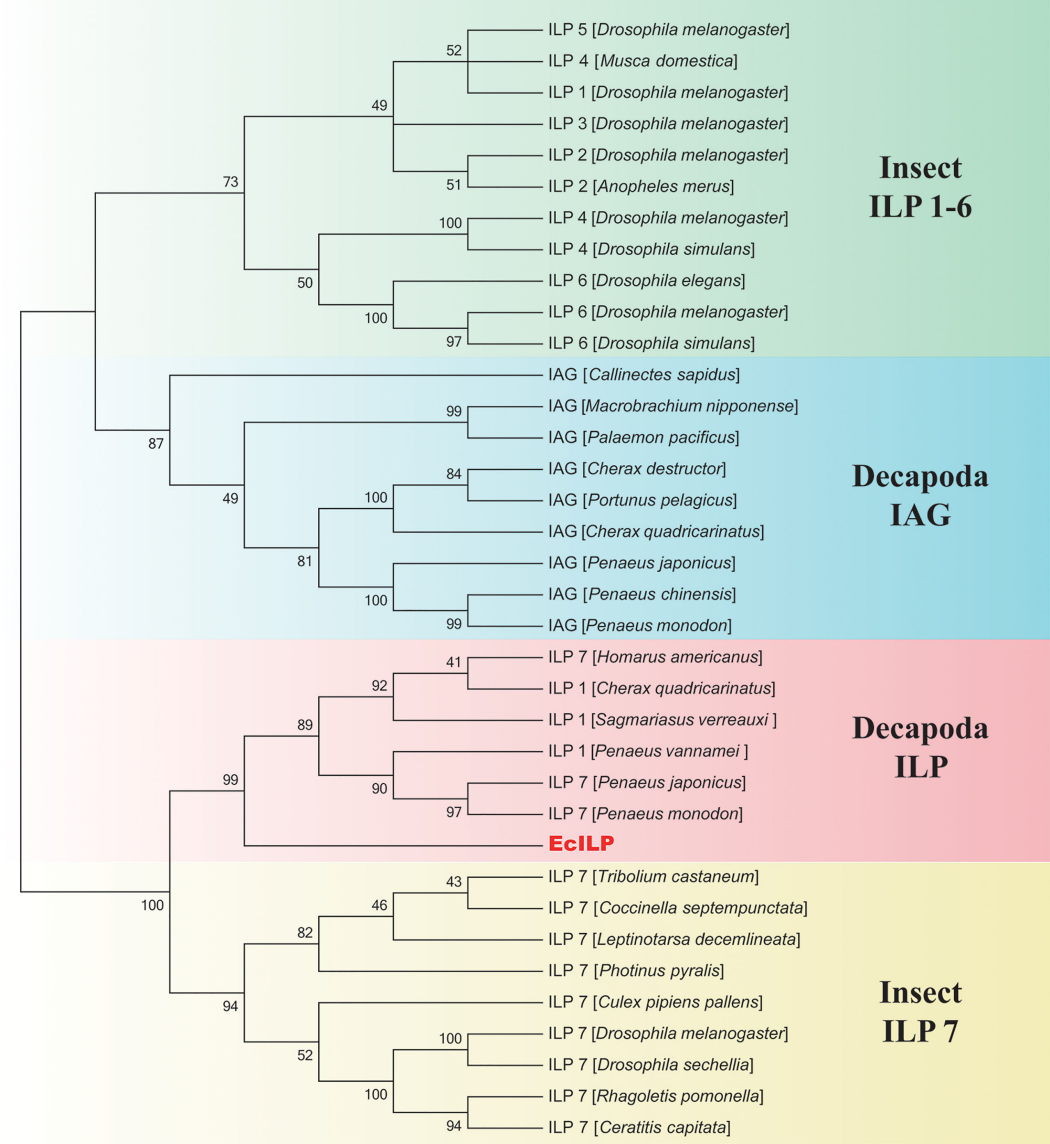
Phylogenetic relationships of insulin-like superfamily members in arthropod. The numbers on the forks are the bootstrap proportions for each branch. The phylogenetic tree was constructed using neighbor-joining (NJ) approach with 1,000 bootstrap replicates.

Phylogenetic analyses showed that ILPs in arthropods could be divided into two main branches, the one clustered decapod ILPs and insect ILP7 together, and another one included decapod IAGs and insect ILP1-6 (**Figure 3**). In the clade of invertebrate ILP7, EcILP clustered with ILP7 homologs of other crustaceans and separated from other ILP members of insects. Among crustaceans, EcILP shared the highest sequence similarity (56.52%) to ILP7 of American lobster *Homarus americanus*, while the sequence similarity rose to 74.68% in mature peptides form.

### *EcILP* gene characterization and sgRNA design

**Figure 4:**
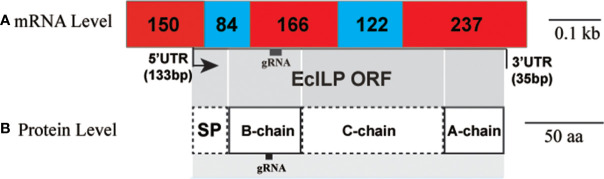
EcILP putative structures. The target site for gRNA is indicated by a black frame. **(A)** Linear model of EcILP mRNA structure. Numbers represent the length of exons. **(B)** Linear model of EcILP amino acid sequence. The correspondence between mRNA structure and amino acid sequence is indicated by one-to-one shade.

To design single-stranded guide RNA (sgRNA), the genomic structure of *EcILP* was assessed by mapping the sequenced ORF to the genomic sequence, which indicated that *EcILP* contained five exons and four introns (**Figure 4A**). Deduced peptide strands (signal peptide, B chain, C chain, and A chain) occupies two, two, three and one exons, respectively (**Figure 4B**). SMART and Pfam analyses revealed the presence of the ILGF domain in mature peptide form of EcILP. The sgRNA is designed to target the B-chain and the third exon of *EcILP* gene. Meanwhile, the sgRNA targets the first cysteine residues for linking the tertiary structure and in front of the ILGF domain. This design ensures that the disruption caused by CRISPR have the greatest impact on its gene structure after knockout.

### Expression profiles of *EcILP*

**Figure 5:**
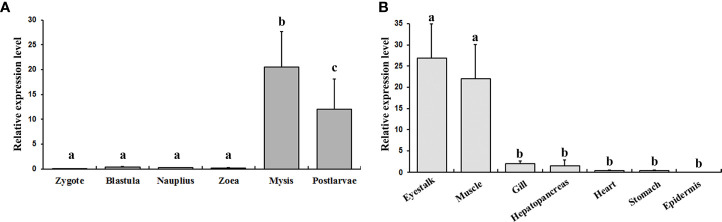
*EcILP* expression profiles at different larval stages **(A)** and different tissues of the adults **(B)**. X-axis shows the different larval stages or adult tissues. Y-axis shows the relative mRNA expression level. Error bars in each column indicate standard deviation, and different letters on the bars indicated the difference significance (*P* value < 0.05). Early developmental samples were collected as pools, and the tissue expression analysis was collected from twelve adult *E. carinicauda*.

The expression profiles of *EcILP* at six larval stages and seven adult tissues were examined by real-time qPCR. The results showed that the *EcILP* mainly expressed at mysis and postlarvae stage at the larval stages (**Figure 5A**). Among all adult tissues, *EcILP* was mainly expressed in the eyestalk and muscle (**Figure 5B**). Statistical analysis showed that the expression level of mysis and postlarvae stage were significantly higher than other stages. Similarly, the expression levels of eyestalk and muscle were significantly higher in adult tissues than in other tissues.

### Generation of CRISPR mutants targeting the *EcILP*

**Figure 6:**
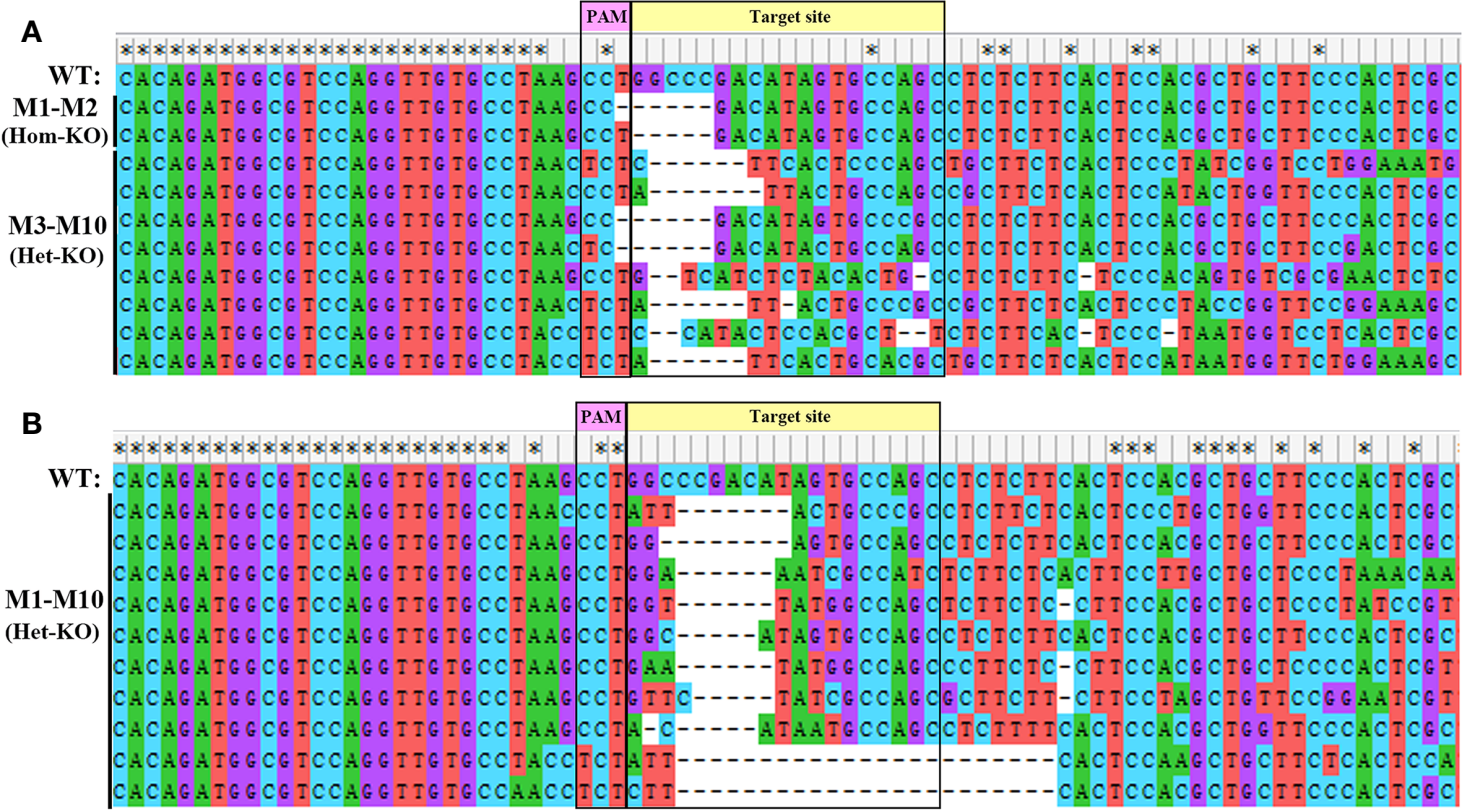
Ten different types of deletion mutants generated in *EcILP*-injected embryos at nauplius stage **(A)** and zoea stage **(B)**. Purple rectangles represent protospacer adjacent motif (PAM) sequences. The yellow rectangle is the 20 bp genomic target site. Deletions are indicated by dashed lines. Bases under asterisks represent fully conserved bases. WT, wild-type. Types of homozygous and heterozygote deletions are marked. The corresponding sequencing chromatograms are shown in **Figure S1** and **S2**.

To explore the physiological functions of *EcILP* during growth of *E. carinicauda*, CRISPR/Cas9 gene-editing technology was applied to generate *EcILP*-knockout (KO) mutants. Gene editing efficiency was detected at 72 h (nauplius stage), 10 days (zoea stage) and 15 days (mysis stage) after microinjection. The sequencing results of PCR amplification products showed that the genomes of *E. carinicauda* in the experimental group were successfully edited (**Figure 6**). The corresponding sequencing chromatograms confirmed the appearance of multiple peaks after the protospacer adjacent motif (PAM) sites (**Figures S1, S2**). Editing efficiency was 17.5% at nauplius stage, 9.3% at zoea stage and 8.7% at mysis stage (**Table 2**). Notably, about 20% of homozygous mutations were detected at the nauplius stage larvae (**Figure 5A**), whose sequencing results showed that both alleles were edited, whereas no homozygous deletions were detected at the zoea and mysis stages (**Table 2** and **Figure 6B**).

**Table 2:** Statistics on the survival and editing efficiency after injection of CRISPR/Cas9 system.

Target genes	Injection	Nauplius stage			Zoea stage			Mysis stage		
	Injected embryos	Surviving embryos	Edited	Homozygous	Surviving embryos	Edited	Homozygous	Surviving embryos	Edited	Homozygous
***EcILP***	~2700	590 (21.9%)	42/240 (17.5%)	9/42 (21%)	300 (11.1%)	14/150 (9.3%)	0%	46 (1.7%)	4/46 (8.7%)	0%
**Control**	~1000	516 (51.6%)	–	–	285 (28.5%)	–	–	135 (13.5%)	–	–

### Analysis of phenotypic changes in *E. carinicauda* after microinjection

The survival rate of injected embryos was detected after microinjection. At the embryonic stage, embryos injected with the *EcILP*-CRISPR system had a significant increased mortality. In the control group, the survival rate reached 51.6% at nauplius stage and 28.5% at zoea stage and the hatching rate was 13.5% at mysis stage. While for the eggs injected with *EcILP*-CRISPR system, the survival rate was 21.9% at nauplius stage and 11.1% at zoea stage, and the hatching rate was only 1.7% at mysis stage. It was found that most individuals injected with *EcILP*-CRISPR system died before hatching.

**Figure 7:**
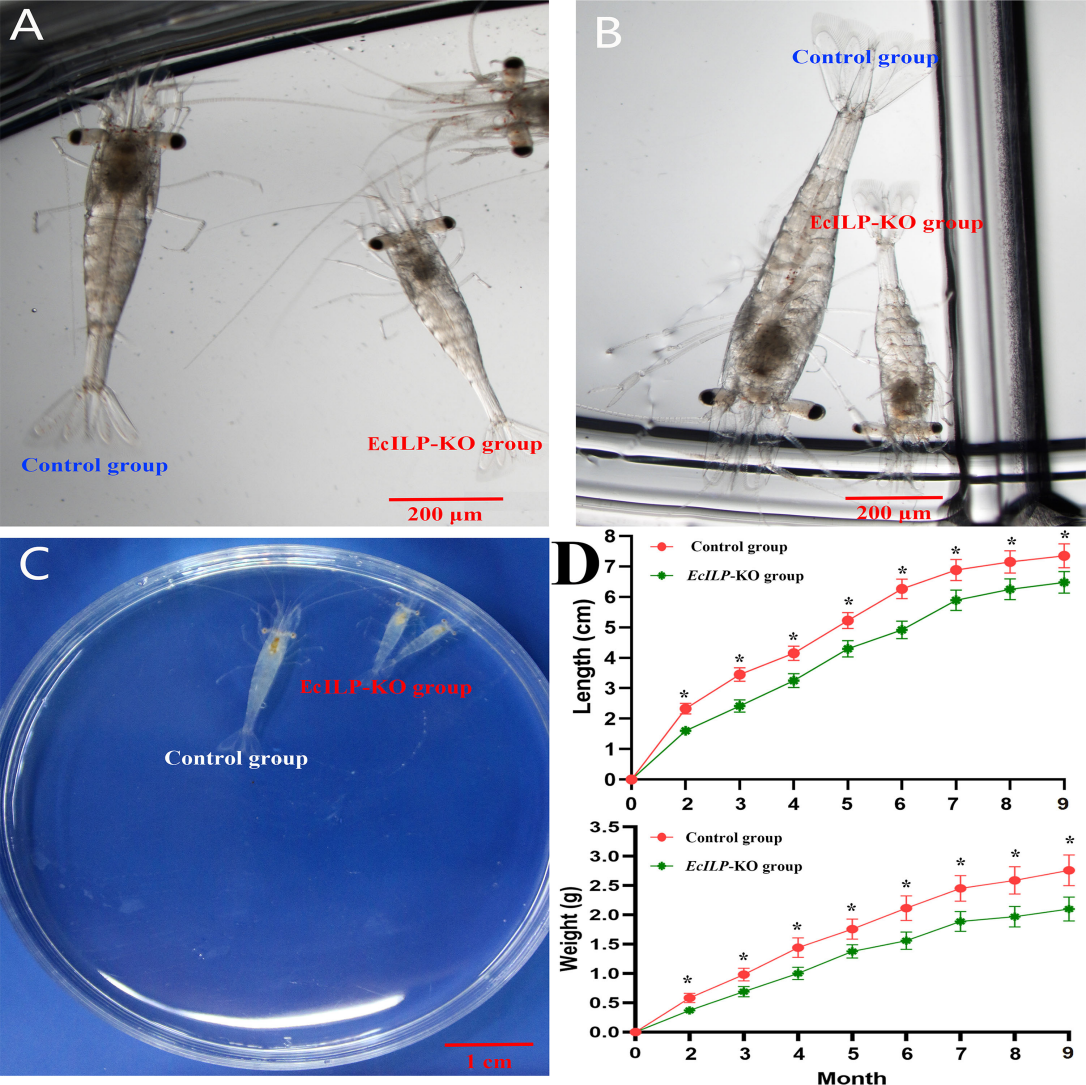
Growth comparison of *EcILP*-KO and control group individuals. **(A)** 18 days after hatching, **(B)** 22 days after hatching, **(C)** One month after hatching, **(D)** Statistical changes in body length and body weight from 2 to 9 months after hatching. Error bars refer to the standard deviation. *P* values are denoted: *P < 0.05.

During the adult stage, we evaluated the growth of *EcILP*-KO individuals from 2 to 9 months after hatching, including the body length and body weight. It showed that the *EcILP*-KO individuals exhibited a remarkable growth-inhibitory phenotype (**Figure 7**). Through statistics analysis on the individuals for nine months growth after hatching, both body weight and body length of *EcILP*-KO individuals were significantly lower than those from the control group (**Figure 7D**).

### Effects of *EcILP* silencing on growth

**Figure 8:**
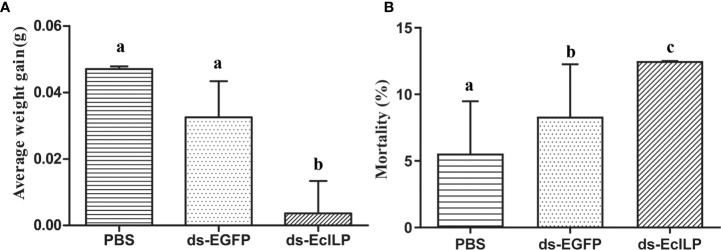
Growth performance of *E. carinicauda* under different treatments. **(A)** average weight gain, **(B)** mortality rate (%). Error bars refer to the standard deviation. Different letters on the bars indicated the difference significance (*P* value < 0.05). Each group (PBS group, ds-EGFP group, and ds-EcILP group) used 75 adult *E. carinicauda*.

In pre-experiment of RNAi, the expression of *EcILP* in the ds-*EcILP* RNA injected group was significantly reduced (*P* < 0.01) to 13.2% of the control group at an interfering concentration of 1 µg/µL. In the RNAi experiment, after silencing *EcILP* with dsRNA for 15 days, the average weight gain of the ds-*EcILP* group was significantly lower than those of the PBS control and ds-*EGFP* groups (**Figure 8A**). Moreover, the mortality rate of ds-*EcILP* group was significantly higher than that of PBS control group and ds-*EGFP* group. The mortality rate of ds-*EcILP* group reached 12.5%, while the mortality rate of control group and ds-*EGFP* group was only 5.56% and 8.33%, respectively (**Figure 8B**).

## Discussion

### The role of ILP in the regulation of crustacean growth

Although most decapod crustaceans are important economic species, studies on the growth regulation mechanism of decapod crustaceans are still limited (41). Whether an insulin-regulated growth pathway exists in decapods has been a mystery, as information on crustacean ILPs has long been limited to IAG, which is considered as a sex-determination factor (42). Recently, application of next-generation sequencing and bioinformatics analysis revealed the presence of more ILPs identified in decapod crustaceans (16, 43), however, the function of these of ILPs (besides IAG) in crustacean are still limited. Moreover, there is a lack of direct evidence for the role of ILPs in crustacean growth regulation (41). In this study, the identification of *EcILP* confirmed the presence of another type (DILP7/relaxin-type) of ILP in the decapod crustacean, *E. carinicauda*, further suggesting the integrity of four types of ILP in crustaceans. Most important, this study provided us with clear evidence that the ILPs may have a growth-regulatory role in crustaceans, and its knockout and interference could significantly affect the growth of prawn, especially for the early development. It is showed that knockout of *EcILP* significantly reduced early larval survival rate by more than half compared with the control group. Similar results were found in other arthropod larvae, such as *Maruca vitrata, Leptinotarsa decemlineata* and *Antheraea pernyi* (44–46), that knockout of ILPs significantly affected larval growth and reduced larval size. During adult development, knockout of ILPs or interfering ILPs expression has been shown to affect adult growth in some arthropods, such as *Drosophila* and *B. mori* (25, 47–49), which is similar to our results that *EcILP* interference can significantly reduce the body weight and survival rate of *E. carinicauda*. *EcILP* findings suggest that there is an association between ILPs and growth in *E. carinicauda.* Furthermore, it further implicates the existence of the insulin-regulated growth pathway in crustaceans (50). Overall, elucidating the function of *EcILP* will help us understand the mechanisms underlying growth regulation in crustaceans.

### The broad conservation of DILP7/relaxin-type ILP

Increasing evidences showed that invertebrate ILPs share substantial sequence and biological functional similarities with mammalian insulins and IGFs (51, 52). The present study further confirmed the functional conservation of DILP7/relaxin-type ILP superfamily in crustaceans from the following aspects. (a) Conserved sequence features. *EcILP* has typical preproinsulin sequence features, including non-conserved signal peptide and C-chain, conserved B-chain, A-chain, cleavage sites, and six conserved cysteines. This indicated that *EcILP* can be activated by the removal of the C peptide and forming a disulphide bridge in the B-A chain, with an additional intra-chain bridge within the A-chain. This knowledge revealed that all proteins that make up the decapod crustacean ILP processing machinery are similar to those of vertebrates, just as conservative sequence characteristics exist in vertebrate IGFs and insect ILPs. (b) Conserved expression pattern. *EcILP* is mainly expressed in the adult eyestalk and muscle. This expression pattern is similar to that of *S. verreauxi* (*Sv-ILP1*), the first identification of DILP7/relaxin-like ILP in crustacean (15), and *Litopenaeus vannamei* (*LvILP7*), which found DILP7/relaxin-like ILP can maintain hemolymph glucose homeostasis for prawn (53). The expression pattern of *EcILP* further suggests that DILP7/relaxin-type ILP is a broad expressed gene in crustacean (15). Moreover, the highest expression signature in the eyestalk further suggests that DILP7/relaxin-like ILP may synthesized in the neuroendocrine system (15). (c) Conserved functional features. As one of the most important hormone growth factors, the growth-regulatory role of the insulin superfamily has been demonstrated in a variety of animals (8, 22, 54). In this study, the apparent growth-inhibitory phenotypes by both CRISPR/Cas9 and RNAi approaches suggested that *EcILP* was involved in the growth of *E. carinicauda*. These data further confirmed the conservation of *ILP* function in invertebrate growth. In *Drosophila*, there was a corresponding increase in body size in adult flies when DILP7 was overexpressed (26), and the pupal volume was significantly smaller in female when DILP7 was knocked down (55). In *Crassostrea gigas*, ILP7 is thought to participate in the energy metabolism and involve in the growth of oysters (56). From an evolutionary perspective, this widespread occurrence and conservation of ILPs across various taxonomic phyla clearly demonstrate the importance of the insulin-signaling pathway across animal kingdom.

### *EcILP* knockout/silencing may affect the survival of *E. carinicauda*

Of the *EcILP*-CRISPR injected embryos, 78.1% and 88.9% of the injected embryos died at the nauplius and zoea stage, respectively, and the hatching rate dropped to 1.7% at mysis stage. The survival rate and hatching rate were significantly lower than those of the control group. Compared with previously reported gene editing studies of *E. carinicauda*, embryos injected with *EcILP*-CRISPR system also had a much higher mortality rate (34, 39, 57, 58). Moreover, it is worthy to note that we failed to detect any homozygous deletions after the zoea and mysis stages, whereas the early homozygous mutation rate was 20%, which indicating the potential lethality of homozygous knockout of *EcILP*. Moreover, the body weight and body length of *EcILP*-KO individuals were significantly lower than those of the control group in growth statistics for nine months. Corresponding RNAi experiment in adults also found that interference of *EcILP* expression significantly reduce the growth rate and increased mortality of *E. carinicauda*. The above results further indicate that *EcILP* is involved in the development and growth of *E. carinicauda*, and is particularly important for early development, and the knockout/silencing of *EcILP* may affect the survival of *E. carinicauda*.

This may be related to the ancient evolutionary position and broad functionality of ILP7. The conservation of ILP7 has been previously reported in the *Drosophila* ILP family, as it is by far the most conserved among the eight DILPs and the only peptide with distinctive orthologues in other species, such as ILP5 in *Anopheles* sp. and ILP7 in *T. castaneum* (10). The emergence of the ILP7 is thought to be traced to a more ancient common ancestor of deuterostomes and protostomes (8, 10, 15). This ancient evolutionary position implies that DILP7/relaxin-like ILP may have more important and broader functions (15). In insects, DILP7/relaxin-like ILP is produced by by three pairs of abdominal neurons and function to regulate growth, appetite and reproduction (25, 26, 59, 60). Moreover, Imambocus *et al.* analyzed the involvement of DILP7 in escape behavior of *Drosophila* and found that DILP7 might be involved in regulating the innate behavior of escape, and silencing of DILP7 neurons strongly impaired larval light avoidance (61). In addition, ILP7 has been reported to play a critical role in lipid metabolism and reproduction in *Aedes aegypti* (23). In crustacean, we analyzed the role of *LvILP7* in maintaining hemolymph glucose homeostasis in *L. vannamei*, and found that interfering with the ILP7 gene could inhibit the clearance of exogenous glucose and affect hemolymph glucose regulation in shrimp (53). Some studies believe that as an ILP7 orthologue, DILP7/relaxin-type ILPs serve a more obvious function in the crustaceans (15). This ancient evolutionary position and broad functions (e.g. growth, metabolism, appetite, and behavior) of DILP7/relaxin-type ILP may determined its importance for crustacean, and thus *EcILP* knockout and interference affect the growth of *E. carinicauda*.

## Conclusion

This study firstly confirmed the existence of DILP7/relaxin-like ILP in the decapod crustacean *E. carinicauda*, whose structure, origin and function are well conserved. This is the first time to characterize the function of DILP7/relaxin-type ILP in crustaceans, filling the research gap of ILPs in the regulation of growth processes in these species. The significant growth-inhibitory phenotypes of *EcILP* knockout/silencing by CRISPR/Cas 9 and RNAi suggested that *EcILP* may affect the normal growth and survival of *E. carinicauda*, which indicated ILP should be an important growth regulator of crustaceans. The results reported in this study provide valuable information for understanding the molecular mechanisms of growth regulation in decapod crustaceans.

## Data availability statement

The datasets presented in this study can be found in online repositories. The names of the repository/repositories and accession number(s) can be found in the article/**Supplementary Material**.

## Author contributions

YG and FL conceived and designed the experiments. YG, XZ, CZ and SL performed the experiments. YG wrote the initial draft of the manuscript and prepared all the figures. XZ, JY and FL reviewed and contributed to the writing of the final manuscript. All authors contributed to the article and approved the submitted version.

## Funding

This work was financially supported by National Key R&D Program of China (2018YFD0900404, 2018YFD0901301, 2018YFD0900103), the National Natural Sciences Foundation of China (31702320, 31972782).

## Conflict of interest

The authors declare that the research was conducted in the absence of any commercial or financial relationships that could be construed as a potential conflict of interest.

## Publisher’s note

All claims expressed in this article are solely those of the authors and do not necessarily represent those of their affiliated organizations, or those of the publisher, the editors and the reviewers. Any product that may be evaluated in this article, or claim that may be made by its manufacturer, is not guaranteed or endorsed by the publisher.
