# Supplementary material for: CRISPR/Cas9-mediated mutation on an insulin-like peptide encoding gene affects the growth of the ridgetail white prawn Exopalaemon carinicauda
Source: Front Endocrinol (Lausanne). 2022 Sep 29;13:986491. doi: 10.3389/fendo.2022.986491 (PMC9556898; doi:10.3389/fendo.2022.986491)
Supplement: Supplementary file 1 [file DataSheet_1.docx]

**Figure S1 Sequencing chromatogram of 10 different types of deletion mutants generated in *EcILP*-injected embryos at nauplius stage.** The sequencing chromatogram of PCR products are corresponding to the sequences in figure 6A. The top sequences represents the wild-type (WT) sequences, with the target sites for single-stranded guide RNA (sgRNA) are indicated by red line and the protospacer adjacent motif (PAM) sites are indicated by black frame. The chromatograms show multiple peaks, indicating the occurrence of the mutations.

**Figure S2 Sequencing chromatogram of 10 different types of deletion mutants generated in *EcILP*-injected embryos at zoea stage.** The sequencing chromatogram of PCR products are corresponding to the sequences in figure 6B. The top sequences represents the wild-type (WT) sequences, with the target sites for sgRNA are indicated by red line and the PAM sites are indicated by black frame. The chromatograms show multiple peaks, indicating the occurrence of the mutations.
