# Supplementary figures and images for: CRISPR/Cas9-mediated mutation on an insulin-like peptide encoding gene affects the growth of the ridgetail white prawn Exopalaemon carinicauda
Source: Front Endocrinol (Lausanne). 2022 Sep 29;13:986491. doi: 10.3389/fendo.2022.986491 (PMC9556898; doi:10.3389/fendo.2022.986491)

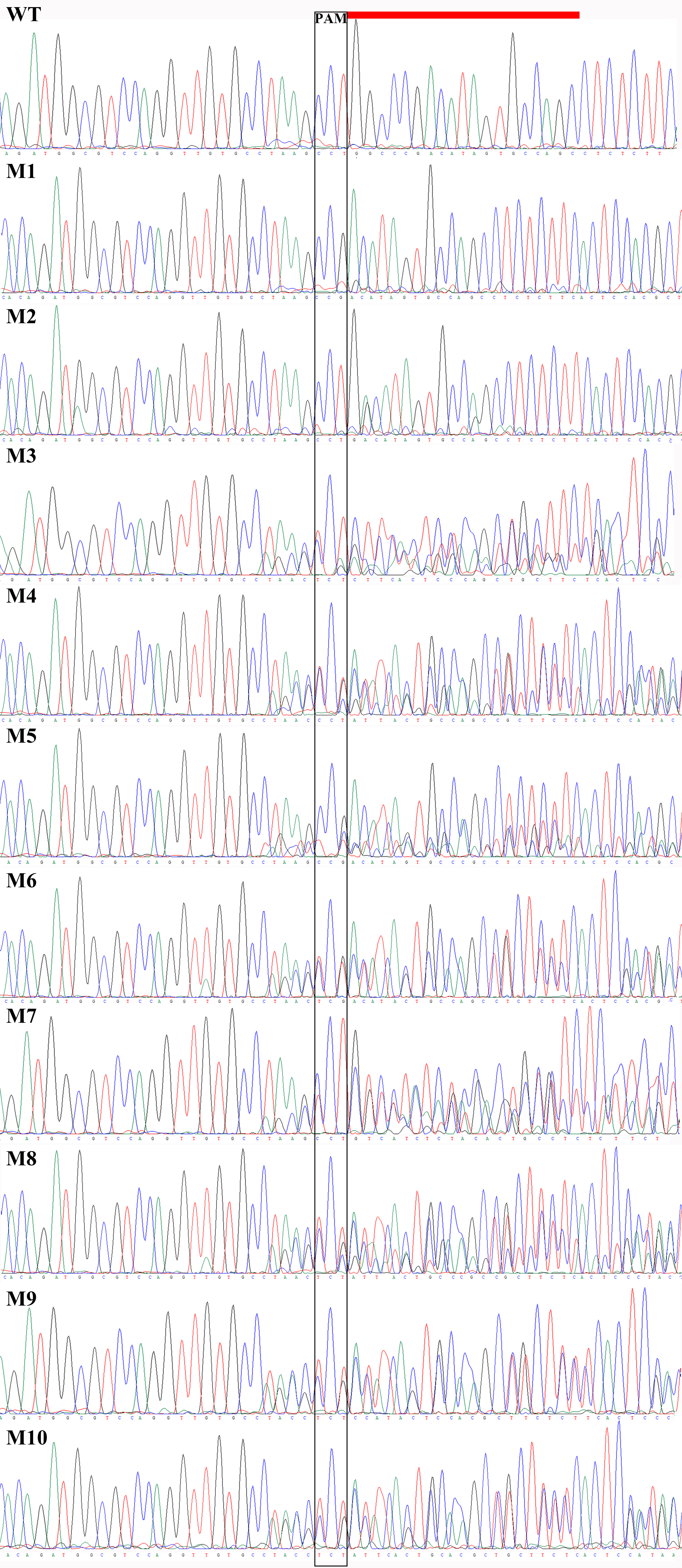

Supplement: Supplementary file 2 [file Image_1.tif]

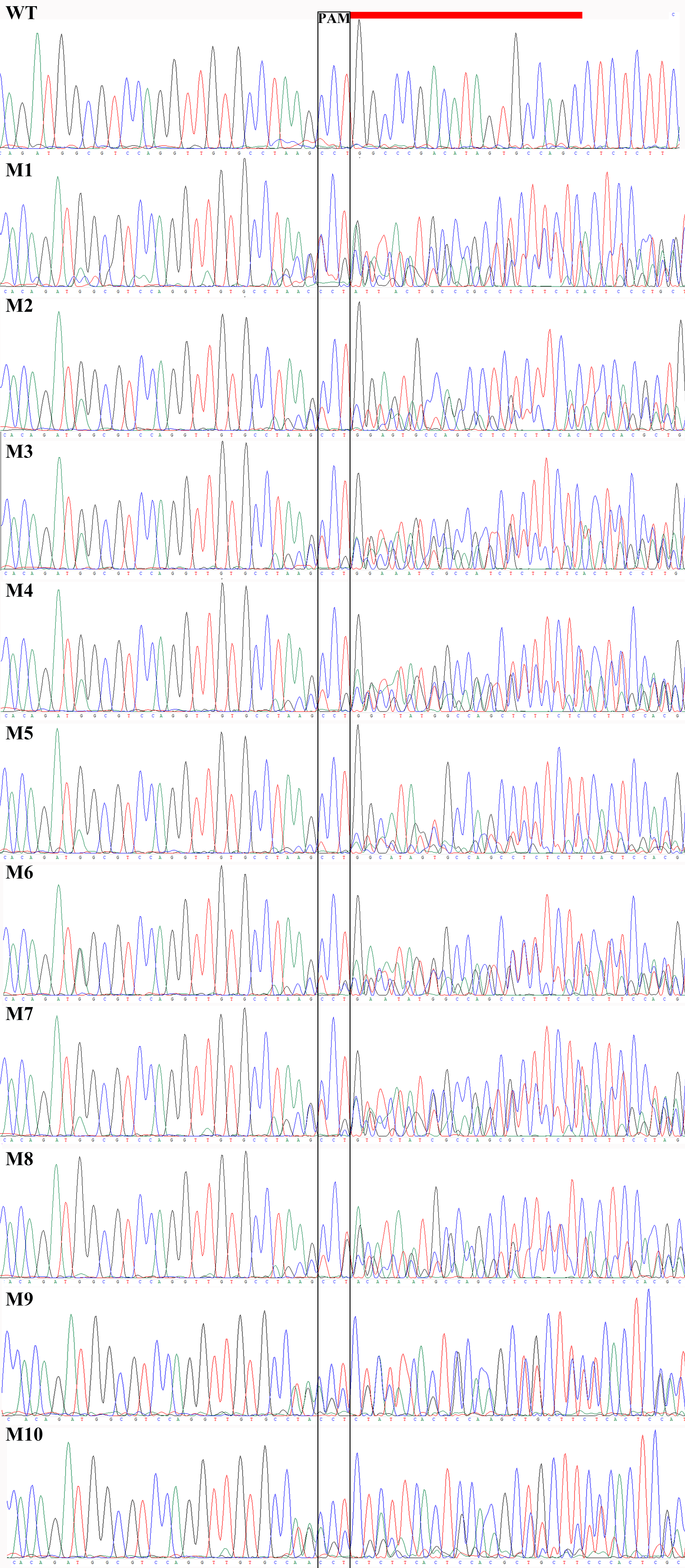

Supplement: Supplementary file 3 [file Image_2.tif]
